# Robust principal component analysis for accurate outlier sample detection in RNA-Seq data

**DOI:** 10.1186/s12859-020-03608-0

**Published:** 2020-06-29

**Authors:** Xiaoying Chen, Bo Zhang, Ting Wang, Azad Bonni, Guoyan Zhao

**Affiliations:** 1 Department of Neuroscience, Washington University School of Medicine, St. Louis, MO USA; 2 Center of Regenerative Medicine, Department of Developmental Biology, Washington University School of Medicine, St. Louis, MO USA; 3 Department of Genetics, Washington University School of Medicine, St. Louis, MO USA; 4 The Edison Family Center for Genome Sciences and Systems Biology, Washington University School of Medicine, St. Louis, MO USA

**Keywords:** Robust principal component analysis, PcaGrid, PcaHubert, Outlier detection, RNA-seq, High-dimensional data, Anomaly detection

## Abstract

**Background:**

High throughput RNA sequencing is a powerful approach to study gene expression. Due to the complex multiple-steps protocols in data acquisition, extreme deviation of a sample from samples of the same treatment group may occur due to technical variation or true biological differences. The high-dimensionality of the data with few biological replicates make it challenging to accurately detect those samples, and this issue is not well studied in the literature currently. Robust statistics is a family of theories and techniques aim to detect the outliers by first fitting the majority of the data and then flagging data points that deviate from it. Robust statistics have been widely used in multivariate data analysis for outlier detection in chemometrics and engineering. Here we apply robust statistics on RNA-seq data analysis.

**Results:**

We report the use of two robust principal component analysis (rPCA) methods, *PcaHubert* and *PcaGrid*, to detect outlier samples in multiple simulated and real biological RNA-seq data sets with positive control outlier samples. *PcaGrid* achieved 100% sensitivity and 100% specificity in all the tests using positive control outliers with varying degrees of divergence. We applied rPCA methods and classical principal component analysis (cPCA) on an RNA-Seq data set profiling gene expression of the external granule layer in the cerebellum of control and conditional *SnoN* knockout mice. Both rPCA methods detected the same two outlier samples but cPCA failed to detect any. We performed differentially expressed gene detection before and after outlier removal as well as with and without batch effect modeling. We validated gene expression changes using quantitative reverse transcription PCR and used the result as reference to compare the performance of eight different data analysis strategies. Removing outliers without batch effect modeling performed the best in term of detecting biologically relevant differentially expressed genes.

**Conclusions:**

rPCA implemented in the *PcaGrid* function is an accurate and objective method to detect outlier samples. It is well suited for high-dimensional data with small sample sizes like RNA-seq data. Outlier removal can significantly improve the performance of differential gene detection and downstream functional analysis.

## Background

In statistics, an outlier is an observation that lies outside the overall pattern of a distribution [1]. However, it is difficult to determine how much different a value has to be in order to be considered an outlier. It becomes increasingly difficult or impossible to determine outliers in multivariate and high-dimensional data. Robust statistics is designed to detect the outliers by first fitting the majority of the data and then flagging data points that deviate from it (i.e. the majority of the data) [2]. Therefore, robust statistics provides automatic ways of detecting and flagging outliers so that outliers can be downweighted or removed, removing the need for manual inspection if desired.

High throughput mRNA sequencing, known as RNA-seq [3], has emerged as a powerful approach of transcriptome profiling to detect genes differentially expressed (DEGs) between two experimental groups. The protocols and data analyses of RNA-seq are relatively mature [4] and have become a powerful tool routinely used in research laboratories. However, RNA-seq experiments are elaborate procedures with many steps, which include mRNA isolation, reverse transcription, fragmentation, adding adapter sequences, PCR, sequencing, etc. Variations in reagents, supplies, instruments and operators may introduce random or systematic errors at any step of the process. We refer these variations as “batch effects” or “unwanted variations”. True biological differences or technical failures during the process of sample preparation could lead to extreme deviation of a sample from samples of the same treatment group (biological replicates). We refer to these samples as “outliers”. Accurate detection of differentially expressed genes (DEGs) depends on accurate estimation of the sample variance. Technical outliers contribute unnecessary variance and lead to decreased statistical power and need to be removed. However, removing biological outliers will result in underestimation of the natural biological variance and will increase the risk of spurious conclusions. Therefore, accurate identification of outliers and careful evaluation of the nature of each outlier are important for later analysis. It has been shown that both “batch effects” and technical “outliers” can be detrimental to the quality of the data and hence affect downstream analyses [5–7].

Research of outlier sample detection in RNA-seq data analysis has been scarce in the literature. Because of the importance of quality control at sample level, outlier sample detection has been studied extensively in microarray data analysis [8–14]. However, methods developed for microarray data sets may not be suitable for RNA-seq data sets because the measurement of the number of sequenced fragments that map to the transcripts in RNA-seq is fundamentally different from gene probe-based methods as in microarray [15]. In addition, comparing to microarray data sets with sample sizes in the tens, or sometimes hundreds or thousands, most RNA-seq studies are relatively small. Due to sample availability and time or cost constraints, it is usually not practical nor is it always possible to have large number of biological replicates for each condition. The most cost effective number of biological replicates is recommended to be 2–6 [16, 17] which is what used in most RNA-seq studies. Scientists have only recently begun to explore the methodology for RNA-seq outlier sample removal [5, 18]. Lopes et al. proposed an ensemble outlier detection approach to identify abnormal cases and consensus covariates from 1222 samples which is not applicable to RNA-seq data with only few biological replicates. The method developed by Norton et al. is specifically designed for differential splicing analysis. In a recent paper of best practices for RNA-seq data analysis, Conesa et al. [4] stated “Reproducibility among technical replicates should be generally high (Spearman R^2^ > 0.9), but no clear standard exists for biological replicates”. The authors argued that if gene expression differences exist among experimental conditions, it should be expected that biological replicates of the same condition will cluster together in a principal component analysis (PCA) [4]. Currently, “visual inspection” of the PCA biplot of principal components 1 and 2 to determine outlier samples is the standard of the field [4, 6]. However, this approach lacks statistical justification and sometime may be difficult to determine or carry unconscious biases.

Robust Principal Component Analysis (rPCA) is designed to use robust statistics to detect outliers objectively, rather than subjectively as currently carried out using classical PCA (cPCA) [2]. cPCA is commonly used for dimension reduction when faced with high-dimensional data. cPCA constructs a set of uncorrelated variables, which correspond to eigenvectors of the sample covariance matrix. However, cPCA is highly sensitive to outlying observations. Consequently, the first components are often attracted toward outlying points, and may not capture the variation of the regular observations. Therefore, data reduction based on cPCA becomes unreliable if outliers are present in the data [19]. rPCA refers to a group of methods that entail robust statistical analysis to (1) obtain principal components that are not substantially influenced by outliers and (2) to identify outliers and determine their category. In recent years, many rPCA algorithms have been developed for high-dimensional data [20, 21] to detect those anomalous observations. There are four major algorithms: *PcaCov*, *PcaGrid*, *PcaHubert* and *PcaLocantore* (see methods for details). All of them are implemented in the *rrcov* R package as different functions with a common interface for computation and visualization [22]. rPCA statistics have been widely used in multivariate data analysis for outlier detection in chemometrics and engineering [19, 23]. To our best knowledge, this is the first application of robust statistics on RNA-seq data analysis.

In this study, we explore the feasibility of using rPCA statistics on RNA-seq data sets with small sample sizes for outlier detection. Previous studies have shown that ROBPCA (*PcaHubert*) outperforms several robust PCA tools [19] and that ROBPCA has the highest sensitivity whereas *PcaGrid* method has the lowest estimated false positive rate [24]. We therefore chose these two methods in our study. We demonstrated that PcaGrid can accurately detect outlier samples from multiple simulated as well as real biological RNA-seq data sets with 100% sensitivity and 100% specificity. Using the real RNA-seq data, we compared eight data analysis strategies including before and after outlier removal as well as with and without batch effect modeling. In addition, we performed quantitative reverse transcription PCR (qRT-PCR) to validate the DEGs and used these results as references to evaluate the performance of the eight data analysis strategies. Our results demonstrated that removing outliers resulted in significantly increased performance in DEGs detection and led to deep insights of underlying gene regulatory mechanisms which could not be revealed before removing outliers.

## Methods

### Animals

Mice carrying SnoN conditional knockout specifically in granule neuron precursors of the mouse cerebellum were generated by crossing *SnoN*^loxp/loxp^ mice with a transgenic mouse line in which the expression of the recombinase Cre is driven by the *Math1* promoter as described [25]. Mice were maintained under pathogen-free conditions. All animal experiments were carried out according to protocols approved by the Animal Studies Committee of Washington University School of Medicine and in accordance with the National Institutes of Health guidelines.

### RNA-seq data simulation

Polyester [26] was used for RNA-seq data simulation with a mean fragment length of 50 bases. The mouse GRCm38/mm10 transcript file downloaded from ENSEMBLE (https://useast.ensembl.org/index.html) was used as input. RNA transcripts with varying degree of coverage and fold change between two conditions were simulated to mimic real biological situations. For baseline samples we specified fold change matrix to have 500 differentially expressed genes between two conditions. Three biological replicates each (*n* = 3 each, group = 2), six or twelve biological replicates each (*n* = 6 or 12 each, group = 2) were simulated using error model of “Illumina 4”, positional bias model of “rnaf” with default error rate of 0.005. Error rate is a simulation parameter which represents the probability that the sequencer records the wrong nucleotide at any given base in the uniform error model. To simulate samples with higher error rates we used the same parameters as those used for the baseline sample simulation except that the error rates were varied from 0.01, 0.05, 0.1 to 0.2. These experiments replicate noises introduced by sequencing errors.

To simulate outlier samples, we designed two type of outliers. The first type of outliers had a distinct set of genes that were differentially expressed between two conditions than the baseline sample set and were referred as outlierH (Outliers with high “outlierness”). This scenario represents experiments where samples were coming from a completely different population. For example, individuals with a wrong diagnosis for the disease of interest. The second type of outliers had 50% DEGs overlapped with that of the baseline sample set but with different fold changes and were referred as outlierL (Outliers with low “outlierness”). This scenario represents experiments where outlier samples were coming from individuals with a correct diagnosis but in the severe end of the disease spectrum or respond differently to a drug treatment. Three biological replicates each for each group were simulated using error model of “Illumina 4”, positional bias model of “rnaf” and error rate of 0.01, 0.05, 0.1 and 0.2.

### RNA-Seq data analysis

The RNA-Seq data set (Gene Expression Omnibus database accession number GSE120279) were generated by laser capture microdissection followed by RNA-Seq. It was used to profile specifically gene expression of the external granule layer in the cerebellum of control and conditional *SnoN* knockout mice (*n* = 6 each group) [27]. RNA-Seq data of Naïve Dorsal Root Ganglia (DRG) neurons obtained from CAST/Ei mouse (GSM1639804) [28] and human cerebellum (GSM2693449) [29] were downloaded from Gene Expression Omnibus database.

Sequences were adapter-trimmed using Cutadapt [30], quality-controlled using PRINSEQ [31] and aligned to mouse genome GRCm38/mm10 using STAR [32]. Reads in features were counted using HTSeq [33]. Genes with less than 10 reads in all samples were excluded from further analysis. DESeq2 [34] were used for DEGs detection and variance stabilizing transformation (*vst*) or regularized log (*rlog*) normalization. *P*-values were adjusted for multiple testing with the Benjamini and Hochberg method for controlling of the false discovery rate (FDR). Genes were called significant when the adjusted P-value was < 0.1. All homologous genes with identical gene names between the human and mouse were used in the human cerebellum data analysis as described [35].

### Outlier detection

There are four major algorithms and all of them are implemented in the *rrcov* R package as different functions with a common interface for computation and visualization [22]. In addition, an outlier map (diagnostic plot) can be generated based on the score distances and orthogonal distances computed for each observation which is especially useful for examining outlying observations. The four major algorithms are: 1) the PCA based on robust covariance matrix estimation algorithms is implemented in the function *PcaCov*; 2) the PCA projection pursuit algorithm is represented by the function *PcaProj* and *PcaGrid* [36]; 3) the ROBPCA algorithm [19, 37] implemented in the *PcaHubert* function tries to combine the advantages of the above two approaches; 4) the spherical principal components algorithm [38] is implemented in the *PcaLocantore* function.

We chose to use *PcaGrid* and *PcaHubert* in our study because of their superior performance comparing to other methods [19, 24]. The *PcaGrid* function implemented the PCA projection pursuit algorithm [36]. It is based on finding projections of the data which have maximal dispersion using the grid search algorithm. Instead of using the variance as a measurement of dispersion, a robust scale estimator is used for the maximization problem [36]. The ROBPCA algorithm [19, 37] implemented in the *PcaHubert* function combines ideas of projection pursuit and robust covariance estimation. The projection pursuit part is used for the initial dimension reduction and the minimum covariance determinant estimator are then applied to this lower-dimensional data space.

**Fig. 1:**
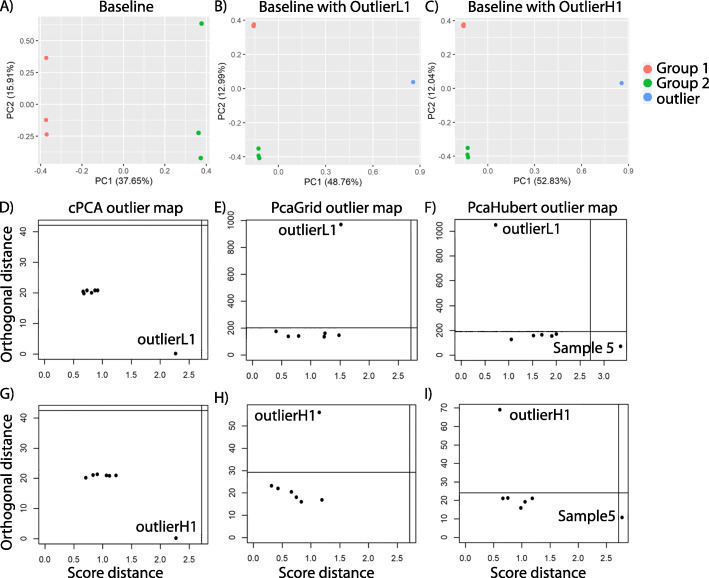
Comparing the performance of cPCA and rPCA on the simulated data. **a** cPCA plot of the simulated baseline data with two treatment groups and 3 biological replicates each. The first principal component captured the variation of the baseline samples between the two groups. **b** cPCA plot of the simulated baseline data plus outlierL1; The first principal component was attracted by outlierL1. **c** cPCA plot of the simulated baseline data plus outlierH1; The first principal component was attracted by outlierH1. **d-f** Outlier maps of the simulated baseline plus outlierL1 data set using (**d**) cPCA, (**e**) PcaGrid and (**f**) PcaHubert. (**g-i**) Outlier maps of the simulated baseline plus outlierH1 data set using (**g**) cPCA, (**h**) PcaGrid and (**i**) PcaHubert. OutlierL1: simulated sample L-1 of the low “outlierness” group. OutlierH1: simulated sample H-1 of the high “outlierness” group. Sample 5: the 5th sample of the baseline data set

The purpose of a robust PCA is twofold: (1) to find those linear combinations of the original variables that contain most of the information, even if there are outliers, and (2) to flag outliers and to determine their type [19]. Hubert et al. illustrated the existence of four types of observations after projecting to the lower-dimension PCA subspace and proposed the outlier map to distinguish between regular observations and the three types of outliers for higher-dimensional data [19]. Briefly, to construct an outlier map, the robust score distance of each observation is plotted on the horizontal axis and the orthogonal distance of each observation to the PCA subspace is plotted on the vertical axis. To classify the observations, cutoff value on the horizontal and vertical axes are determined using corresponding distribution to have an exceeding probability of 2.5% [19]. By combining both distance measures the outlier map allows to distinguish between four types of data points. (1) Regular observations form one homogeneous group that is close to the PCA subspace. They have both a small orthogonal distance and a small score distance and are clustered at the bottom left corner of the outlier map (Fig. 1f). (2) The good leverage points lie close to the PCA space but far from the regular observations. They have a high score distance but a small orthogonal distance, such as point Sample 5 in Fig. 1f. (3) Orthogonal outliers have a large orthogonal distance to the PCA space but a small score distance, like point outlierL1 in Fig. 1f. This type of points cannot be distinguished when we look only at their projection on the PCA space. (4) Bad leverage points have both a large orthogonal distance and a large score distance which will be located at the top right corner of the outlier map if existed. They lie far from the PCA subspace spanned by the robust principal components, and after projection on that space they lie far from most of the other projected data.

The count matrix of each sample being tested as an outlier was individually combined with the baseline data matrix or the *SnoN* knockout experiment data matrix. The combined count matrix was used as input to DESeq2 for DEGs detection and *rlog* or *vst* transformation. Transformation normalized matrix with n rows (samples) and p columns (genes) was provided as input to the *PcaHubert* and *PcaGrid* function implemented in the *rrcov* R package for outlier detection. Default parameters were used for the analysis unless specifically mentioned otherwise.

### qRT-PCR validation and statistics analysis

External granule layer granule neuron precursors were purified using traditional Percoll fractionation method as described [39]. Total RNA was extracted from the purified granule cells of each genotype (*n* = 4 or 5) using RNAeasy Micro Kit (Qiagen). cDNA was synthesized from 250 ng of RNA using Superscript III (Invitrogen) with random hexamer and olig-dT combined primers. PCR reactions were performed using lightcycle480 Roche. The primers used in validation are listed in Supplement Table 6. A ratio *t*-test with a predefined nominal α level of 0.05 was used to determine significant differences between *SnoN* KO mice and wild-type littermate controls.

## Results

### rPCA accurately detect outliers from simulated RNA-Seq data

To test whether rPCA can be used to identify outliers, we first tested rPCA on simulated data. We simulated an RNA-Seq data set for two treatment groups with 3 biological replicates each using Polyester [26] and used it as the baseline sample set (Fig. 1a). Next, we simulated 28 RNA-Seq data sets with varying degrees of deviation from the baseline samples (Table 1). Each one was combined with the baseline sample set and used as a negative or positive control outlier in an experiment to determine the accuracy of rPCA in outlier detection.

**Table 1:** Performance of rPCA for outlier detection on simulated data with 3 biological replicates in each treatment group using *rlog* transformation

ID of sample being added	outlier model	Error rate	Sample replicate	Outlier detected by PcaHubert	Number FP outlier called by PcaHubert	outlier detected by PcaGrid	Number FP outlier called by PcaGrid	SEN (%) by PcaGrid	SP (%) by PcaGrid
None (baseline)	NA	0.005		NA	NA	NA	NA	NA	NA
N-1	NA	0.01	1	NA	0	NA	0	NA	NA
N-2		0.05	1	NA	1	NA	0	NA	NA
N-3		0.1	1	NA	1	NA	0	NA	NA
N-4		0.2	1	NA	1	NA	0	NA	NA
L-1	outlierL	0.01	1	Yes	1	Yes	0	100	100
L-2		0.01	2	Yes	1	Yes	0	100	100
L-3		0.01	3	Yes	1	Yes	0	100	100
L-4		0.05	1	Yes	1	Yes	0	100	100
L-5		0.05	2	Yes	1	Yes	0	100	100
L-6		0.05	3	Yes	1	Yes	0	100	100
L-7		0.1	1	Yes	1	Yes	0	100	100
L-8		0.1	2	Yes	1	Yes	0	100	100
L-9		0.1	3	Yes	1	Yes	0	100	100
L-10		0.2	1	Yes	1	Yes	0	100	100
L-11		0.2	2	Yes	1	Yes	0	100	100
L-12		0.2	3	Yes	1	Yes	0	100	100
H-1	outlierH	0.01	1	Yes	1	Yes	0	100	100
H-2		0.01	2	Yes	1	Yes	0	100	100
H-3		0.01	3	Yes	1	Yes	0	100	100
H-4		0.05	1	Yes	1	Yes	0	100	100
H-5		0.05	2	Yes	1	Yes	0	100	100
H-6		0.05	3	Yes	1	Yes	0	100	100
H-7		0.1	1	Yes	1	Yes	0	100	100
H-8		0.1	2	Yes	1	Yes	0	100	100
H-9		0.1	3	Yes	1	Yes	0	100	100
H-10		0.2	1	Yes	1	Yes	0	100	100
H-11		0.2	2	Yes	1	Yes	0	100	100
H-12		0.2	3	Yes	1	Yes	0	100	100

*SEN* Sensitivity, *SP* Specificity, *rlog* Regularized log transformation, *vst* Variance Stabilizing Transformation, *outlierL* Outlier with low “outlierness”, *outlierH* Outlier with high “outlierness”

We first simulated RNA-Seq data using the same parameters that were used for the baseline sample simulation except for the different error rate varying from 0.01 to 0.2 to serve as negative controls. *PcaGrid* did not report any outlier for any of the data set tested whereas *PcaHubert* reported one outlier in 3 out 4 of the cases when *rlog* was used for normalization (Table 1, sample N-1to N-4). When *vst* was used for normalization *PcaHubert* reported one (in 3 out 4 of the cases) or two (1 case) baseline samples as outliers (Supplemental Table 1, sample N-1to N-4). This is consistent with previous finding that *PcaGrid* method has the lowest estimated false positive rate [24].

We then simulated RNA-Seq data with low “outlierness” comparing with that of the baseline samples and used each sample as a positive control outlier (Table 1 and Supplemental Table 1, sample L1 to L12). The first two principal components of cPCA on the count matrix of the first simulated experiment (sample L-1 of outlierL group as the positive control outlier, hereby referred to as outlierL1) normalized using the *rlog* function in the DESeq2 package are shown in Fig. 1b. The first component was attracted toward the outlying point and failed to capture the variation of the baseline samples because of the existence of the outlying observation (comparing Fig. 1a, b). Visual inspection of the PCA plot suggested that outlierL1 is an outlier. However, the outlier map of cPCA failed to classify it as an outlier (Fig. 1d). *PcaGrid* correctly identified the outlier sample without reporting any false positive outlier for any of the data sets tested when *rlog* (12 out of 12 cases, Fig. 1e, Table 1) or *vst* (12 out of 12 cases, Supplemental Table 1) was used for normalization. In addition to correctly identifying the outlier sample, *PcaHubert* reported one baseline sample as an outlier in 12 out of 12 cases when *rlog* was used for normalization (Fig. 1f, Table 1), indicating false positives. Similarly, when *vst* was used for normalization, *PcaHubert* reported one (11 out of 12 cases) or two baseline samples (one case) as outliers (Supplemental Table 1). Again, this is consistent with a previous finding that *PcaGrid* method has the lowest estimated false positive rate [24]. More false positive outliers were reported by *PcaHubert* when *vst* was used for normalization than when *rlog* transformation was used. *Rlog* is more robust and sometimes performs qualitatively better than the *vst* which is useful when checking for outliers (https://github.com/mikelove/DESeq2/blob/master/R/rlog.R).

Furthermore, we simulated RNA-Seq data with high “outlierness” comparing with that of the baseline samples and used each sample as a positive control outlier (Fig. 1c, Table 1 and Supplemental Table 1, sample H1 to H12). Similarly, cPCA outlier map failed to identify the outlier even though visual inspection of the PCA biplot suggested that outlierH1 is an outlier sample. *PcaGrid* correctly identified the outlier sample without reporting any false positive outlier at all error rates tested ranging from 0.01 to 0.2 for *rlog* normalized data (Fig. 1g-i, Table 1). Again, *PcaHubert* reported one baseline sample as an outlier in every experiment indicating false positives (Fig. 1i, Table 1, Supplemental Table 1). More false positive outliers were reported by *PcaGrid* when *vst* was used for normalization (Supplemental Table 1) than when *rlog* transformation was used (Table 1).

Lastly, to test whether rPCA can be applied to data set with more samples, we simulated an independent RNA-Seq data set with 6 biological replicates each for two treatment groups as the baseline sample set and repeated the above experiments. Consistent with the above results, using *PcaGrid* method and *rlog* for normalization we achieved 100% sensitivity and 100% specificity for outlier detection in all simulated tests with varying degrees of “outlierness” (Supplemental Table 2). When *vst* was used for normalization *PcaGrid* reported one false positive outlier in 4 experiments out of 24 total experiments (Supplemental Table 3). *PcaHubert* reported one false positive outlier in every experiment in both *rlog* and *vst* normalized data (Supplemental Table 2 and 3). We further simulated an independent RNA-Seq data set with 12 biological replicates each for two treatment groups as the baseline sample set and repeated the above experiments. Again, using *PcaGrid* method and *rlog* for normalization we achieved 100% sensitivity and 100% specificity for outlier detection in all simulated tests with varying degrees of “outlierness” (Supplemental Table 4). When *vst* was used for normalization both methods reported 2 false positive outliers for all experiments that only negative control outlier were used. *PcaGrid* reported 1 or 2 false positive outliers in 8 experiments out of 24 total experiments with positive control outliers (Supplemental Table 5). *PcaHubert* reported 3 false positive outliers in every experiment in both *rlog* and *vst* normalized data (Supplemental Table 4 and 5). In summary, using *PcaGrid* method and *rlog* for normalization we can achieve 100% sensitivity and 100% specificity for outlier detection in all simulated tests with varying degrees of “outlierness”.

### The effect of parameter values on outlier detection using simulated RNA-Seq data

In the above analysis, we used default values for all the parameters required by *PcaHubert* and *PcaGrid* function. To understand how different parameter values affect the results we performed systematic test of diagnostic parameters using one sample from each outlier model as a positive control outlier (Table 2).

**Table 2:** The effect of parameter values on outlier detection using simulated data

Outlier added	Outlier model	Error rate	Method	Parameter name	Default (range)	Parameter value	Outlier called	Number FP outlier called
L-1	outlierL	0.01	PcaGrid	crit.pca.distances	0.975	≤ 0.844	Yes	> 1
						0.845–0.912	Yes	1
						0.913–0.999	Yes	0
						1	No	0
			PcaHubert	crit.pca.distances	0.975	≤ 0.643	Yes	> 1
						0.643–0.982	Yes	1
						0.983–0.999	Yes	0
						1	No	0
				alpha	0.75 (0.5–1)	0.5–0.683	Yes	1
						0.684–0.749	Yes	> 1
						0.750–0.999	Yes	1
						1	No	0
H-1	outlierH	0.01	PcaGrid	crit.pca.distances	0.975	≤ 0.648	Yes	> 1
						0.649–0.750	Yes	1
						0.751–0.999	Yes	0
						1	No	0
			PcaHubert	crit.pca.distances	0.975	≤ 0.669	Yes	> 1
						0.669–0.978	Yes	1
						0.979–0.999	Yes	0
						1	No	0
				alpha	0.75 (0.5–1)	0.5–0.714	Yes	0
						0.715–0.999	Yes	1
						1	No	0

*crit.pca.distances* is the criterion used for computing the cutoff values for the orthogonal score distances. The default value is 0.975 for both *PcaHubert* and *PcaGrid* functions. *PcaHubert* behaved similarly on outlierH and outlierL. Any value below 0.643 (outlierL) or 0.669 (outlierH) lead to multiple false positives, any value between 0.643–0.982 (outlierL) or 0.669–0.978 (outlierH) resulted in one false positive, and any value between 0.983–0.999 (outlierL) or 0.979–0.999 (outlierH) lead to the correct identification of the outlier without any false positives. A value of 1 or above was not able to identify any outlier. The value of *crit.pca.distances* required to correctly identify the outlier without any false positives was a very narrow range and the default value was too low in both cases.

*PcaGrid* behaved very differently on outlierH and outlierL. A sample with low “outlierness” (outlierL) required higher cutoff values (0.913–0.999) to be identified correctly without calling false positives than when a sample had high “outlierness” (outlierH, 0.751–0.999). The default value of *crit.pca.distances* required to correctly identify the outlier without any false positives was appropriate in both situations. *PcaGrid* had more accurate and stable performance than *PcaHubert* for a wider range of parameter values for this data set.

*PcaHubert* has an additional parameter *alpha,* which measures the fraction of outliers the algorithm should resist. *Alpha* can take any value between 0.5 and 1 and the default is 0.75. For samples with low “outlierness” (outlierL), all allowed values lead to the correct identification of the outlier as well as calling false positive outliers. For samples with high “outlierness” (outlierH), certain values will allow the correct identification of the outlier without calling false positives (0.5–0.714) but the default value (0.75) is not an appropriate cutoff.

In conclusion *PcaGrid* had wider range of parameter values with stable performance and the default cutoff value allowed the correct identification of outliers without yielding false positives in most tested cases. The default cutoff values for *PcaHubert* was too low which allowed the correct identification of outliers but also led to false positive calls.

### rPCA accurately detects outliers from composite real RNA-Seq data

To mimic true biological situations where outlier samples exist, we used three published data sets and performed analysis where samples of different sources (different species or different tissues) were mixed with the samples of interest. The first data set was generated by laser capture microdissection followed by RNA-Seq to profile specifically gene expression of the external granule layer (EGL) in the cerebellum of control and conditional *SnoN* knockout mice [27]. The cerebellum from postnatal day 6 mice was dissected. Laser capture microdissection was used to isolate specifically the EGL layer of the cerebellum. EGL isolated from six conditional *SnoN* knockout mice and six littermate control mice were subjected to RNA-Seq. This data set was sample of interest and we henceforth refer to this as the mouse cerebellum data set. Samples were collected from two litters and processed in 4 batches with litter and batch matched controls (Table 3). We reported detected DEGs and functional analysis of DEGs in the previous publication [27]. Here we report the data analysis method and the qRT-PCR validation results. RNA-Seq data of human cerebellum (GSM2693449) [29] and Naïve Dorsal Root Ganglia (DRG) neurons from CAST/Ei mouse (GSM1639804) [28] were downloaded from Gene Expression Omnibus and used as positive control outliers. Expression matrix were generated by combining the count matrix of the corresponding positive control outlier sample (*n* = 1) with that of the mouse cerebellum data (WT *n* = 6, KO n = 6). The combined data matrices were referred as human cerebellum data set and DRG neuron data set, respectively.

**Table 3:** Mouse cerebellum data set sample information

Samples	Gender	Batch	RIN	Littermates
KO-1	M	1	7.8	1/10/2018
WT-1	M	1	7.3	1/10/2018
KO-2	M	1	7.4	1/10/2018
WT-2	M	1	8.4	1/10/2018
KO-3	F	2	7.2	1/10/2018
WT-3	F	2	8.1	1/10/2018
KO-4	F	3	8.1	1/15/2018
WT-4	F	3	8.5	1/15/2018
KO-5	M	3	7.6	1/15/2018
WT-5	M	3	8.2	1/15/2018
KO-6	F	4	7.5	1/15/2018
WT-6	F	4	8.1	1/15/2018

*RIN* an RNA integrity number for assigning integrity values to RNA measurements

**Fig. 2:**
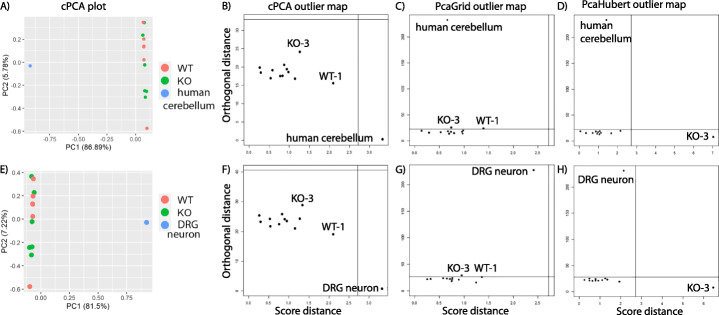
Comparing the performance of cPCA and rPCA on the composite real RNA-Seq data of the human cerebellum dataset (**a-d**) and the mouse dorsal root ganglion (DRG) neuron dataset (**e-h**). cPCA plot (**a**) and outlier maps for human cerebellum dataset using cPCA(**b**), *PcaGrid* (**c**) and *PcaHubert* (**d**). cPCA plot (**e**) and outlier maps for the mouse dorsal root ganglion (DRG) neuron dataset using cPCA(**f**), *PcaGrid* (**g**) and *PcaHubert* (**h**)

As expected, the first component was attracted toward the outlying human cerebellum sample using cPCA (Fig. 2a). Visual inspection of the PCA biplot indicate that human cerebellum sample was an outlier and the outlier map of cPCA correctly classified it as an outlier (Fig. 2b). Both *PcaHubert* and *PcaGrid* correctly identified the human cerebellum sample as an outlier using default parameters (Fig. 2c, d) demonstrating that rPCA can accurately distinguish samples from different species. Interestingly, both methods also classified a sample from *SnoN* knockout mice (KO-3) as an outlier. *PcaGrid* classified an additional sample from control animals (WT-1) as an outlier. To test whether rPCA can distinguish samples from the same species but from different tissues, we applied the three methods on the DRG neuron dataset. Similarly, all three methods correctly classified the mouse DRG neuron sample as an outlier using default parameters (Fig. 2e-h). Again, both *PcaHubert* and *PcaGrid* determined *SnoN* knockout sample KO-3 as an outlier and *PcaGrid* also classified control sample WT-1 as an outlier. In these two cases, by visual inspection alone outliers can be correctly identified using cPCA and the outlier map agreed with the visual inspection. As expected, rPCA accurately identified true positive control outliers from the two composite real RNA-Seq data. However, *SnoN* knockout sample KO-3 was classified as an outlier by both *PcaHubert* and *PcaGrid* methods in both the human cerebellum and the mouse DRG neuron data set. To better understand the data we next analyzed the *SnoN* knockout cerebellum dataset using *PcaHubert* and *PcaGrid* without artificial outlier added.

### Comparing the performance of cPCA and rPCA on real RNA-Seq data

**Fig. 3:**
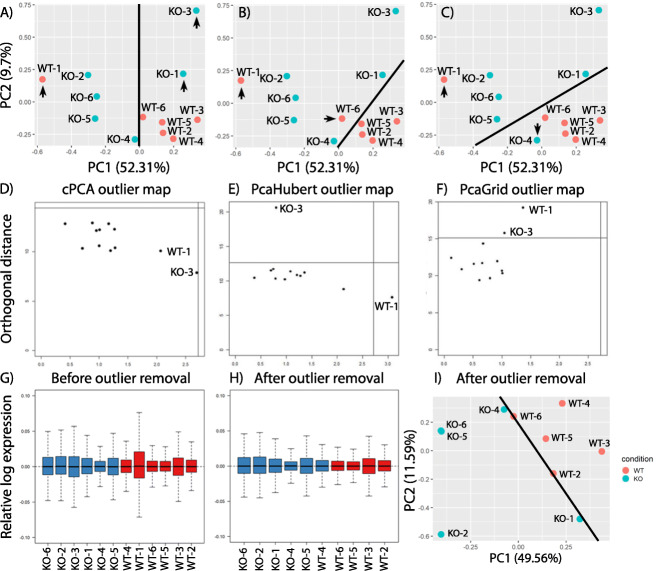
Comparing the performance of cPCA and rPCA on the mouse cerebellum data. **a-c** cPCA plot of the mouse cerebellum data set with three possible scenarios to achieve separation between groups: (**a**) removal WT-1, KO-1 and KO-3; (**b**) removal WT-1 and WT-6; (**c**) removal WT-1 and KO-4. Arrows point to candidate outlier samples need to be removed. **d-f** Outlier maps of the mouse cerebellum data set using (**d**) cPCA, (**e**) PcaHubert and (**f**) PcaGrid. **g** Relative log expression (RLE) plot of the mouse cerebellum data before removing outliers WT-1 and KO-3, (**h**) RLE plot after removing outliers and (**i**) cPCA plot after removing outliers. Black line in (**a, b, c, i**) indicates the line that separates WT and *SnoN* KO samples

To compare the performance of cPCA and rPCA, each method was applied to the mouse cerebellum data count matrix normalized using the *rlog* function in DESeq2 package. The first two principal components of cPCA are shown in the scatter plots of Fig. 3a-c. Visual inspection of the PCA plot suggested that there are at least three possible scenarios that can achieve two-group separation with a minimal number of outlier samples removed. If we assume principle component 1 is the major contributor that separates WT and *SnoN* KO then WT-1, KO-3 and KO-1 should be removed as shown in Fig. 3a (option 1). Alternatively, we can remove WT-1 and WT-6 (Fig. 3b, option 2) or WT-1 and KO-4 (Fig. 3c, option 3) to achieve the separation of WT and *SnoN* KO samples if both principle component 1 and 2 are affected by the phenotype. Outlier map of classical PCA did not identify any outlier sample (Fig. 3d) and cPCA could not provide statistics to distinguish outlier sample(s) from the normal samples. Therefore, researchers are left to define outliers based on heuristics that may not be accurate or carry unconscious biases no matter which option is chosen.

On the contrary, rPCA classified samples WT-1 and KO-3 as outliers based on robust statistics. The same *rlog* normalized count matrix of the mouse cerebellum data set was used as input to *PcaHubert* and *PcaGrid*. Both *PcaHubert* and *PcaGrid* classified KO-3 and WT-1 as outliers using default parameters (Fig. 3e, f) without additional samples identified as outliers. Removing these two samples led to reduced sample heterogeneity (Fig. 3g, h) and better separation of *SnoN* knockout and control sample (Fig. 3i). After careful examination of experimental records, sample KO-3 was found to had been left in a freezer chamber for an hour before sectioning which may be the factor that contributed to the significant deviation of the sample from the rest of samples. We were not able to identify the factor that contributed to the variation of sample WT-1 which reflected a common situation in real biological experiments.

In summary, rPCA detected samples significantly diverged from samples of the same treatment group due to technical failure. *PcaGrid* and *PcaHubert* identified the same two samples as outliers in the mouse cerebellum data set whereas cPCA failed to detect any.

### The effect of outliers on DEG detection in real RNA-Seq data

To determine how the presence of outliers affected DEG detection in real RNA-Seq data analysis, we compared DEG detection before and after outlier removal in the *SnoN* knockout cerebellum data set. Because samples were processed in 4 batches we modeled batch and RIN effect (~ batch + RIN + condition) or modeled only batch effect (~ batch + condition) or only RIN (~ RIN + condition) in the design formula of DESeq2. Together we compared a total of eight strategies of data analysis:

Before outlier removal:

Strategy 1 (S1): no batch effect modeling.

Strategy 2 (S2): modeling of only batch effect in DESeq2.

Strategy 3 (S3): modeling of only RIN in DESeq2.

Strategy 4 (S4): modeling of both batch effect and RIN in DESeq2.

After outlier removal:

Strategy 5 (S5): no batch effect modeling.

Strategy 6 (S6): modeling of only batch effect in DESeq2.

Strategy 7 (S7): modeling of only RIN in DESeq2.

Strategy 8 (S8): modeling of both batch effect and RIN in DESeq2.

**Fig. 4:**
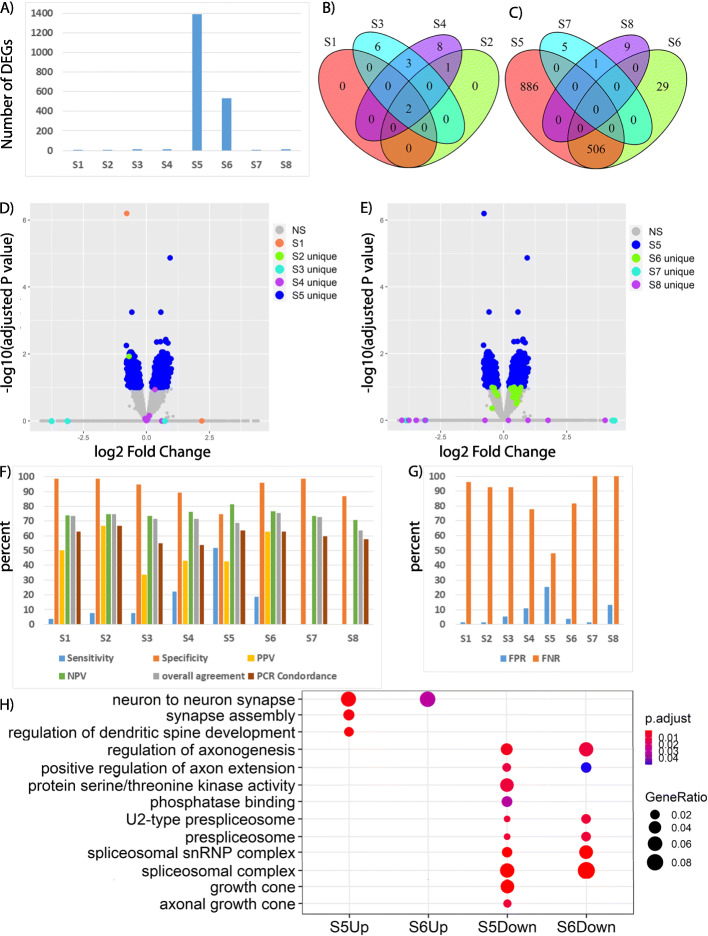
Comparison of DEG detection performance of the eight different data analysis strategies. **a** Number of DEGs; **b** Overlap of DEGs for the four strategies before outlier removal; **c** Overlap of DEGs for the four strategies after outlier removal; **d** Volcano plot of gene expression changes using S5. The x axis specifies the log2 fold-changes, and the y axis specifies the negative logarithm to the base 10 of the adjusted *p* values. Blue dots represent genes expressed at significantly higher (*n* = 792) or lower (*n* = 600) levels upon *SnoN* loss, respectively (adjusted *p* < 0.1). DEGs identified by S1(2), DEGs identified by S2 but not S1 (1), DEGs identified by S3 but not S1, S2 (9) and DEGs unique to S4 (8) and DEGs identified by S5 were labeled with respective colors; **e** Volcano plot of gene expression changes using S5 as described in panel (**d**); DEGs identified by S5, DEGs unique to S6 (29), DEGs identified by S7 but not S8 (6) and DEGs unique to S8 (8) were labeled with respective colors. Using qRT-PCR results as the reference standard we compared (**f**) sensitivity, specificity, positive predictive value (PPV), negative predictive value (NPV), PCR concordance, overall agreement between DESeq2 and qRT-PCR results and (**g**) false positive rate (FPR) and false negative rate (FNR) of the eight strategies. **h** Selective gene ontology (GO) terms from GO enrichment analyses of genes that determined to be up-regulated by S5 (S5Up), S6 (S6Up) or down-regulated by S5 (S5Down) and S6 (S6Down) in conditional *SnoN* KO samples. Before outlier removal: S1: Strategy 1; S2: Strategy 2; S3: Strategy 3; S4: Strategy 4; After outlier removal: S5: Strategy 5; S6: Strategy 6; S7: Strategy 7; S8: Strategy 8

Before outlier removal, four different analysis strategies (S1-S4) detected only 2 to 14 DEGs (Fig. 4a, b). After outlier removal, S5 resulted 1392 DEGs (Fig. 4a). Majority of the DEGs identified by S1-S4 did not overlap with S5 DEGs (Fig. 4d). S6 identified 535 DEGs and 506 of them overlapped with the S5 DEGs (Fig. 4a, c, e). Both S5 and S6 were significant improvement over all strategies before removing outliers. However, S7 and S8, modeling of RIN only or modeling of both batch and RIN effect in the design formula of DESeq2 after outlier removal, resulted only 6 and 10 DEGs respectively (Fig. 4a, c). Interestingly, the 6 and 10 DEGs did not overlap with any DEGs detected by S5 or S6 strategies (Fig. 4c, e) and all the DEGs have very high log2 fold change (value range: 17–30) and high standard error of the log2 fold change. Therefore, including RIN into the design formula for differential expression analysis masked DEGs from being identified. In summary, removing outliers (S5, S6) led to significant increase of number of DEGs as compared with number of DEGs detected by using strategies without outlier removal (S1 – S4).

### Outlier removal enabled the detection of true biologically relevant DEGs

Even though outlier removal led to significantly more DEGs, it remained to be determined whether those DEGs represented true biological differences between *SnoN* KO mice and their littermate controls. To compare the performance of different analysis strategies, we performed qRT-PCR validations of DEGs identified using different data analysis strategies. To provide independent validation of the RNA-Seq results, we used an alternative method -- the traditional Percoll fractionation method [39], to isolate EGL cells from independent biological replicates instead of using the original laser micro-dissected samples. We selected 102 genes for qRT-PCR validation including all the DEGs from S1, S2, S4 and S8 as well as randomly selected DEGs and non-DEGs from S3, S5, S6 and S7 (Supplement Table 6). qRT-PCR was performed in four or five biological replicates for each genotype. Genes were compared to the endogenous control GAPDH, which did not significantly change between the two genotypes. A ratio *t*-test with a predefined nominal α level of 0.05 was used to determine significant differences between *SnoN* KO mice and wild-type littermate controls. By this criteria, 27 genes were significantly different between WT and *SnoN* KO granule neuron precursors. *SnoN*, the gene targeted for conditional knock out, was most significantly differentially expressed by qRT-PCR measure and was determined to be differentially expressed by strategies S2, S4, S5 and S6. We used qRT-PCR result as the reference standard and compared the performance of each strategy with qRT-PCR results.

First, we calculated sensitivity, specificity, positive predictive value, negative predictive value, overall agreement, concordance with qRT-PCR results, false positive rate and false negative rate of the DEG prediction by different strategies. The most dramatic change after outlier removal was the significant increase of sensitivity from 3.7–22.2% (S1-S4) to 51.9% (S5, Fig. 4f) and concomitant decrease of false negative rate from 77.8–96.3% (S1-S4) to 48.1% (S5, Fig. 4g). Batch effect modeling using the design formula of DESeq2 slightly improved the performance before outlier removal (Fig. 4f-g, comparing S1 and S2–4). Removing outlier alone significantly increased the number of DEGs and sensitivity and reduced the false negative rate (S5). After outlier removal, modeling of batch effect only also significantly increased the number of DEGs. However, the sensitivity, specificity and false negative rate were comparable with results from S1-S4. Modeling RIN only or modeling both batch effect and RIN failed to detect any of the qRT-PCR validated DEGs (Fig. 4f). Because of the large number of genes that were not significantly different between *SnoN* KO and the littermate controls, the specificity, negative predictive value, overall agreement were comparable. Concordance with qRT-PCR results was defined as the direction of fold change comparing *SnoN* KO EGL with that of WT littermate controls was consistent across all qRT-PCR replicates and between DESeq2 data analysis and qRT-PCR results. DESeq2 analysis from the eight strategies had 55–68 genes (53.9–66.7%) with the same trends as the qRT-PCR results. Therefore, outlier removal without using batch effect modeling led to detecting significantly more biological relevant DEGs as validated by qRT-PCR on independent biological replicates prepared using a different cell isolation method than all the other strategies.

In addition, we also examined the biological importance of the detected DEGs. The results were validated and published in a previous publication [27]. S5 identified 1392 DEGs with 600 genes downregulated and 792 genes upregulated in the EGL of conditional *SnoN* KO animals compared with littermate control animals. Gene ontology (GO) analyses of DEGs identified by S5 in conditional *SnoN* KO samples demonstrated that genes upregulated in *SnoN* KO EGL were related to neuronal development, dendrite morphogenesis, and neuron projection whereas genes downregulated in conditional *SnoN* KO mice were associated most notably with the control of cell adhesion, mRNA splicing, and protein phosphorylation [27] (Fig. 4h). In addition, transcription factor binding motif enrichment analysis on the promoter sequences of DEGs led to the identification of two candidate transcription factors, N-myc and Pax6, which may regulate these DEGs. Co-immunoprecipitation analyses demonstrated that SnoN forms physical complexes with N-myc and Pax6 and may thereby regulate programs of cell proliferation and cell differentiation gene expression in granule neuron precursors in the postnatal mammalian brain [27]. Comparison of GO analyses of genes that determined to be up-regulated by S5 (S5Up), S6 (S6Up) or down-regulated by S5 (S5Down) and S6 (S6Down) in conditional *SnoN* KO samples demonstrated that DEGs identified by S5 and S6 are enriched for similar GO terms related to dendrite morphogenesis, mRNA splicing etc. (Fig. 4h). However, some GO terms associated with known *SnoN* function was identified as significantly enriched only for DEGs identified by S5. For example, *SnoN* promotes axonal growth in the cerebellum [40] and axon growth cone associated genes was enriched only in DEGs identified by S5. Similarly, *SnoN* promotes protein phosphorylation [41] and GO term “protein serine/threonine kinase activity” was significant only for DEGs identified by S5 (Fig. 4h).

In summary, outlier removal led to significant improvement of DEG detection and enabled downstream functional analysis to reveal true biological relevant regulatory mechanisms. All the strategies before outlier removal yield very few DEGs and it was not possible to perform downstream functional analysis in this case.

## Discussion

In this study, using simulated and real RNA-Seq data we demonstrated that rPCA provided a statistically sound, impartial analysis to detect outliers from RNA-Seq data sets with small sample sizes. *PcaGrid* with default parameters on *rlog* transformation normalized data achieved 100% sensitivity and 100% specificity for all tests using both simulated and real RNA-Seq data. Removing outliers significantly improved the performance of DEG detection and enabled downstream data analysis that provided biological insights to the gene regulatory mechanism. rPCA should be applicable to other similar high-dimensional genomic data including but not limited to ChIP-seq, DNase-seq, and bisulfite sequencing data.

Identification and careful evaluation of the nature of an outlier is important for biologically meaningful data analysis. In biology, outliers could arise due to errors or technical failures. For example, errors can occur in measurement variation, in data entry, in sampling, in low quality data or because of a failed experiment. Collecting tissue of interest could have various amount of unwanted surrounding tissue contamination. Alternatively, outliers could be genuine extreme values. However, the genuine extreme values could be biologically relevant or could be the result of some hidden variation in experiment which researchers were not aware of. An example of biologically relevant outlier is different outcomes of drug treated cancer patients. Analysis of a few patients with metastatic cancer survive exceptionally longer than others under the same treatment identified multiple common mutations of NOTCH2, NF1, FANCD2, PIK3CB and EPHA5 in tumors that responded exceptionally well to treatments [42]. On the other hand, different batch of reagents can have unexpected consequences on gene expression [17]. Accurate identification of outliers enabled scientists to further investigate the potential factors contributing to the outlierness of the sample and to evaluate the biological importance of the sample. In our situation, we expect all the samples from the wild-type animals to be similar to each other and to be clustered together in the PCA biplot. In particular, in the analysis of *SnoN* mutant cerebellum samples WT-1 and WT-2 were animals of the same sex, from the same litter and were processed in the same batch (Table 3). However, WT-2 was similar to whereas WT-1 was highly diverged from other wild-type samples. Therefore, WT-1 was removed from further analysis even though we were not able to identify the factor that contributed to the variation of sample WT-1.

One of the key findings from this study is *PcaGrid* method achieved 100% sensitivity and 100% specificity in outlier detection for both simulated data with varying degrees of outlierness and for real biological data. This finding demonstrated that *PcaGrid* method is a great tool for unbiased outlier detection and can be easily incorporated into sample quality control workflows. In addition, this study demonstrated that outliers can have a profound effect on differential expression results which is consistent with previous findings [43]. The most prominent effect of outlier on DEG detection is the significant decrease of sensitivity and increase of false negative rate. Downstream functional analysis was possible only after outliers were removed.

Unexpectedly using batch effect modeling of batch and RIN in DESeq2 had varying impact on DEG detection. Before outlier removal, batch effect modeling of batch and RIN individually or in combination slightly improved the number of DEGs and sensitivity but decreased specificity, PPV and overall PCR concordance. On the contrary, after outlier removal, batch effect modeling strategies all significantly reduced the number of DEGs and sensitivity. Modeling only batch (S6) did improve specificity, PPV and overall agreement comparing with S5 but with significantly decreased sensitivity and missing known genes and functions regulated by *SnoN*. In particular, modeling of RIN only or together with batch (S7 and S8) led to completely loss of detecting biologically meaningful DEGs. *SnoN*, the gene that was knocked out in the experiment, was not identified as differentially expressed by S7 and S8. Adjusting batch effects could introduce spurious group differences [44] or remove the true biological signals [45]. Even worse, biological heterogeneity can be mistaken for batch effects and wrongfully removed [46] depending on the experimental design and the nature of the batch effect. In this case, modeling of RIN completely removed biological signals. This is likely due to the correlation of RIN with the biological groups because the RIN ranged 7.4–8.1 for the remaining KO samples, and 8.1–8.5 for the remaining WT samples after outlier removal. Therefore experimental testing using orthogonal methods is important to validate computational findings.

## Conclusions

Our results demonstrate that robust principal component analysis implemented in the *PcaGrid* function of the *rrcov* R package is an accurate method to detect outlier samples from RNA-Seq data sets with small sample sizes. Removing outliers can significantly improve the performance of differential gene detection and downstream functional analysis. Robust statistics have been widely used in multivariate data analysis for outlier detection in chemometrics and engineering. To our best knowledge, this study is the first one to apply robust statistics on RNA-Seq data analysis. rPCA should be applicable to other similar high-dimensional genomic data including but not limited to ChIP-seq, DNase-seq, and bisulfite sequencing data.

## Supplementary information

**Additional file 1. **Supplemental Table 1: Performance of rPCA for outlier detection on simulated data with 3 biological replicates in each treatment group using *vst* transformation.

**Additional file 2. **Supplemental Table 2: Performance of rPCA for outlier detection on simulated data with 6 biological replicates in each treatment group using *rlog* transformation.

**Additional file 3. **Supplemental Table 3: Performance of rPCA for outlier detection on simulated data with 6 biological replicates in each treatment group using *vst* transformation.

**Additional file 4. **Supplemental Table 4: Performance of rPCA for outlier detection on simulated data with 12 biological replicates in each treatment group using *rlog* transformation.

**Additional file 5. **Supplemental Table 5: Performance of rPCA for outlier detection on simulated data with 12 biological replicates in each treatment group using *vst* transformation.

**Additional file 6.** Supplemental Table 6: List of genes validated by qRT-PCR with primer sequences, results and DEG status in each analysis strategy.

## Data Availability

The datasets generated and analyzed during the current study are available in the Gene Expression Omnibus database: RNA-Seq data of the external granule layer in the cerebellum of control and conditional SnoN knockout mice (GSE120279) [27], Naïve Dorsal Root Ganglia (DRG) neurons obtained from CAST/Ei mouse (GSM1639804) [28] and human cerebellum (GSM2693449) [29].

## Abbreviations

PCA: Principal component analysis
cPCA: Classical principal component analysis
rPCA: Robust principal component analysis
DEG: Differentially expressed genes
PCR: Polymerase chain reaction
qRT-PCR: Quantitative reverse transcription PCR
KO: Knock out
WT: Wild type
