# Supplementary material for: Robust principal component analysis for accurate outlier sample detection in RNA-Seq data
Source: BMC Bioinformatics. 2020 Jun 29;21:269. doi: 10.1186/s12859-020-03608-0 (PMC7324992; doi:10.1186/s12859-020-03608-0)
Supplement: Supplementary file 1 — Additional file 1. Supplemental Table 1: Performance of rPCA for outlier detection on simulated data with 3 biological replicates in each treatment group using vst transformation. [file 12859_2020_3608_MOESM1_ESM.docx]

**Supplemental Table 1**: Performance of rPCA for outlier detection on simulated data with 3 biological replicates in each treatment group using *vst* transformation.

| ID of sample being added | outlier model | error  rate | sample  replicate | outlier  detected by  PcaHubert | Number FP outlier called by  PcaHubert | outlier detected by PcaGrid | Number FP outlier called  by PcaGrid | SEN (%)  by PcaGrid | SP (%)  by PcaGrid |
| --- | --- | --- | --- | --- | --- | --- | --- | --- | --- |
| None  (baseline) | NA | 0.005 |  | NA | NA | NA | NA | NA | NA |
| N-1 | NA | 0.01 | 1 | NA | 1 | NA | 0 | NA | NA |
| N-2 |  | 0.05 | 1 | NA | 1 | NA | 0 | NA | NA |
| N-3 |  | 0.1 | 1 | NA | 1 | NA | 0 | NA | NA |
| N-4 |  | 0.2 | 1 | NA | 2 | NA | 0 | NA | NA |
| L-1 | outlierL | 0.01 | 1 | Yes | 1 | Yes | 0 | 100 | 100 |
| L-2 |  | 0.01 | 2 | Yes | 1 | Yes | 0 | 100 | 100 |
| L-3 |  | 0.01 | 3 | Yes | 1 | Yes | 0 | 100 | 100 |
| L-4 |  | 0.05 | 1 | Yes | 1 | Yes | 0 | 100 | 100 |
| L-5 |  | 0.05 | 2 | Yes | 2 | Yes | 0 | 100 | 100 |
| L-6 |  | 0.05 | 3 | Yes | 1 | Yes | 0 | 100 | 100 |
| L-7 |  | 0.1 | 1 | Yes | 1 | Yes | 0 | 100 | 100 |
| L-8 |  | 0.1 | 2 | Yes | 1 | Yes | 0 | 100 | 100 |
| L-9 |  | 0.1 | 3 | Yes | 1 | Yes | 0 | 100 | 100 |
| L-10 |  | 0.2 | 1 | Yes | 1 | Yes | 0 | 100 | 100 |
| L-11 |  | 0.2 | 2 | Yes | 1 | Yes | 0 | 100 | 100 |
| L-12 |  | 0.2 | 3 | Yes | 1 | Yes | 0 | 100 | 100 |
| H-1 | outlierH | 0.01 | 1 | Yes | 1 | Yes | 0 | 100 | 100 |
| H-2 |  | 0.01 | 2 | Yes | 1 | Yes | 0 | 100 | 100 |
| H-3 |  | 0.01 | 3 | Yes | 1 | Yes | 0 | 100 | 100 |
| H-4 |  | 0.05 | 1 | Yes | 1 | Yes | 0 | 100 | 100 |
| H-5 |  | 0.05 | 2 | Yes | 1 | Yes | 0 | 100 | 100 |
| H-6 |  | 0.05 | 3 | Yes | 1 | Yes | 0 | 100 | 100 |
| H-7 |  | 0.1 | 1 | Yes | 1 | Yes | 0 | 100 | 100 |
| H-8 |  | 0.1 | 2 | Yes | 1 | Yes | 2 | 100 | 66.7 |
| H-9 |  | 0.1 | 3 | Yes | 1 | Yes | 0 | 100 | 100 |
| H-10 |  | 0.2 | 1 | Yes | 1 | Yes | 2 | 100 | 66.7 |
| H-11 |  | 0.2 | 2 | Yes | 1 | Yes | 0 | 100 | 100 |
| H-12 |  | 0.2 | 3 | Yes | 1 | Yes | 0 | 100 | 100 |

SEN: sensitivity; SP: specificity; rlog: regularized log transformation; vst: Variance Stabilizing Transformation;
